# A MID-1DC+LRT Multi-Task Model for SOH Assessment and RUL Prediction of Mechanical Systems

**DOI:** 10.3390/s25051368

**Published:** 2025-02-23

**Authors:** Hai Yang, Xudong Yang, Dong Sun, Yunjin Hu

**Affiliations:** 1 School of Mechanical Engineering, Guizhou University, Guiyang 550025, China; haiy@uok.edu.gr; 2 Guizhou Institute of Technology, Guiyang 550003, China; 20130219@git.edu.cn; 3 Key Laboratory of Advanced Manufacturing Technology of the Ministry of Education, Guizhou University, Guiyang 550025, China; yunjinh@uok.edu.gr

**Keywords:** predictive health management, state of health, remaining useful life, multi-task model, low-rank transformer

## Abstract

Predictive health management (PHM) plays a pivotal role in the maintenance of contemporary industrial systems, with the evaluation of the state of health (SOH) and the prediction of remaining useful life (RUL) constituting its central objectives. Nevertheless, existing studies frequently approach these tasks in isolation, overlooking their interdependence, and predominantly concentrate on single-condition settings. While Transformers have demonstrated exceptional performance in RUL prediction, their substantial parameter requirements pose challenges to computational efficiency and practical implementation. Further, multi-task learning (MTL) models often experience performance deterioration as a result of imbalanced weighting in their loss functions. To address these challenges, the MID-1DC+LRT model was proposed in the present study. The proposed model integrates a multi-input data 1D convolutional neural network (1D-CNN) and low-rank transformer (LRT) within an MTL framework. This model processes high-dimensional sensor data, multi-condition data, and health indicator data, optimizing the Transformer structure to reduce computational complexity. A homoscedastic uncertainty-based method dynamically adjusts multi-task loss function weights, improving task collaboration and model generalization. The results demonstrate that the proposed model significantly outperformed existing methods in SOH assessment and RUL prediction under multi-condition scenarios, demonstrating superior prediction accuracy and computational efficiency, especially in complex and dynamic environments.

## 1. Introduction

With the continuous advancement of technology and rapid industrial development, modern engineering systems are becoming increasingly complex, accompanied by heightened demands for reliability and safety. As such, traditional maintenance strategies are insufficient to meet the operational requirements of such intricate equipment. In response, predictive health management (PHM) has emerged as a critical technology in equipment maintenance. The fundamental objective of PHM is to enable real-time assessment of system health and forecast the future performance of equipment, thereby facilitating proactive maintenance and optimized operational management [1,2]. The key to achieving PHM lies in using intelligent sensors for real-time data collection and applying advanced data analysis methods to extract key features of equipment health status. Such features not only support the assessment of state of health (SOH) but also aid in predicting the remaining useful life (RUL), thereby providing a data-driven foundation for maintenance and operational decisions [3]. Effective SOH assessment and RUL prediction are essential for enabling precise maintenance scheduling, minimizing downtime and maintenance costs, and preventing significant safety incidents. Consequently, the development of robust models capable of performing SOH assessment and RUL prediction under complex and variable operating conditions is crucial for enhancing the effectiveness of PHM and optimizing equipment management [4,5].

For SOH prediction of complex mechanical equipment, data-driven methods have gained wide attention due to their ability to achieve high-accuracy RUL prediction without relying on physical failure models [6,7,8,9]. These methods can be broadly classified into three categories: statistical model methods, machine learning methods, and deep neural network methods. Statistical model methods rely on prior knowledge to construct empirical models that describe performance degradation, often depending on the extraction and selection of critical features from sensor data. Machine learning methods, on the other hand, aim to directly establish a mapping relationship between sensor data and RUL, but they often struggle with capturing complex nonlinear relationships, especially when dealing with high-dimensional sensor data, where their performance may be suboptimal. In comparison, deep neural network methods [10,11,12,13], with their superior ability to handle high-dimensional data and autonomously learn features, can construct end-to-end prediction models and have thus gained widespread attention in the field. Additionally, deep neural networks have been successfully applied in other fields [14,15,16,17]. Against this background, numerous researchers [4,18] have introduced data-driven deep learning approaches to enhance the accuracy of RUL prediction. For example, Xu et al. [18] proposed a multi-scale convolutional neural network (CNN) model that demonstrated significant improvements in RUL prediction accuracy for tools by effectively extracting complex features from sensor data. Xu et al. [4] constructed an unsupervised neural network-based multi-scale multi-head attention autoencoder-decoder model (MSMHA-AED) to develop an intelligent health index (HI) model for achieving bearing RUL prediction without manual intervention. Nonetheless, despite advances in deep learning research, most methods are designed for predictions under single-condition scenarios, where the extraction of performance degradation features under multi-condition scenarios often suffers from limited generalization capability, resulting in a marked decline in prediction accuracy. For instance, Al-Khazraji et al. [19] employed an autoencoder-based deep belief network (AE-DBN) for predicting the RUL of engines using the single-condition FD001 dataset from C-MAPSS, achieving root mean square error (RMSE) prediction accuracy that outperformed existing deep learning approaches. However, when applied to the multi-condition FD004 dataset, the prediction accuracy dropped drastically to 43%. This highlights the need for further research into improving the adaptability of such methods for both multi- and single-condition prediction scenarios. To address this issue, Wei et al. [20] proposed a conditional variational Transformer-based architecture for RUL prediction under multi-condition scenarios. This method extracts and integrates key features from condition monitoring data using two generative networks and two predictive networks. Experimental results demonstrated that the conditional variational Transformer architecture could effectively predict the RUL of bearings under multi-condition scenarios in the C-MAPSS dataset. By minimizing Kullback–Leibler (KL) divergence between features extracted in different feature spaces, the predictive model was found to outperform traditional methods on multi-condition data [20]. Moreover, the Transformer architecture offers the capability for inter-task collaborative learning in MTL through its sharing mechanism, effectively mitigating overfitting to single-sensor data and enhancing model stability and reliability across varying conditions. For instance, Zhang et al. [21] introduced a Transformer-based multi-task model designed for SOH evaluation and RUL prediction in engines. By utilizing the Transformer’s attention mechanism, the model achieved exceptional performance in feature extraction. Further, the multitask model’s feature-sharing mechanism enabled collaborative learning between SOH evaluation and RUL prediction, leading to a substantial improvement in prediction accuracy under multi-condition scenarios. Yet, in practical applications, the efficiency of Transformer models in MTL is limited due to their large number of parameters and high computational complexity. To balance the trade-off between computational efficiency and feature extraction capability of Transformers, Winata et al. [22] proposed the low-rank transformer, which significantly reduces computational complexity while maintaining feature extraction ability through matrix decomposition techniques. Experimental results revealed that this method significantly improved computational efficiency with minimal loss of feature extraction capability. Despite such improvement, the application of Transformer-based methods remains primarily concentrated in domains like speech recognition and has yet to be extensively validated in prediction tasks involving complex time series data. Moreover, the optimization of MTL models often depends on empirically determined task weights. Variability in these weights under different working conditions can lead to suboptimal performance for specific tasks. In particular, dynamically adjusting task weights to balance inter-task collaborative optimization and task independence presents a significant challenge in MTL, especially in complex multi-condition scenarios.

The aforementioned deep learning-based methods have demonstrated promising results in RUL prediction, but they still face several challenges when applied to mechanical equipment with complex fault modes and variable operating conditions. Firstly, most studies have emphasized sensor data feature extraction while overlooking the substantial impact of operating condition variations on equipment performance degradation. Operating condition changes often serve as critical factors influencing performance decline, especially in multi-condition environments, where model adaptability and generalization remain constrained. Secondly, many methods struggle to capture both temporal and spatial characteristics of data effectively, leading to suboptimal feature extraction. Although Transformer models exhibit exceptional feature extraction capabilities through their attention mechanisms, their high computational complexity and large parameter requirements pose significant burdens for industrial applications. At the same time, while MTL enhances inter-task collaborative optimization, the empirically determined task weight settings in current approaches often fail to maintain stability under varying conditions in complex multi-task scenarios. To address such limitations, the MID-1DC+LRT model was proposed. The model is an innovative framework combining multi-data inputs, 1D-CNN, low-rank transformers, and an MTL structure. This model aims to deliver an efficient and high-accuracy solution for RUL prediction in complex, multi-task environments. The primary contributions of this research are summarized as follows:

(1) Multi-input data MTL framework: An MTL framework was proposed that integrates sensor data, condition data, and health index data to more comprehensively capture multi-source features under complex operating conditions.
(2) Application of low-rank optimized transformer in time series data: By introducing low-rank transformer technology, the computational complexity of the model is significantly reduced while its ability to extract global features from time series data is enhanced.
(3) Loss function weight adjustment based on homoscedastic uncertainty method: A method based on homoscedastic uncertainty was proposed, which models the uncertainty of task losses to achieve dynamic adjustment of loss function weights. This method adaptively optimizes weight allocation based on the uncertainty levels of different tasks, enhancing the collaboration and independence of MTL while improving the model’s robustness and prediction performance under complex multi-condition scenarios.

The remainder of this paper is organized as follows: Section 2 outlines the research problem and presents the proposed MID-1DC+LRT model. Section 3 details an experimental study conducted using the C-MAPSS dataset to assess the effectiveness of the proposed model. Section 4 concludes the study by summarizing the findings and discussing the limitations of this research.

## 2. Problem Description and Proposed Model

In this section, the research problem is described, and the MID-1DC+LRT in MTL model proposed for SOH evaluation and RUL prediction tasks is introduced.

### 2.1. Problem Description

In modern industrial systems, sensors enable the acquisition of extensive real-time data on equipment operating conditions, including environmental, operational, and degradation data. These multidimensional sensor datasets provide critical information for SOH evaluation and RUL prediction, supporting more accurate and informed maintenance decisions. However, extracting effective degradation features from high-dimensional and complex multi-condition data remains a significant challenge. Existing approaches often fail to adequately capture the impact of condition variations on equipment degradation in multi-condition environments, resulting in insufficient generalization capabilities. Further, feature extraction methods struggle to effectively combine temporal and spatial characteristics, leading to suboptimal utilization of condition data and reduced model adaptability. While Transformers exhibit powerful feature extraction capabilities, their high computational complexity limits their practical application in industrial contexts. Similarly, the reliance on empirically determined task weight settings in MTL hinders the dynamic balancing of optimization demands across tasks, affecting both model stability and overall performance in complex scenarios. To address such issues, the MCI-1DC+LRT model was introduced, leveraging the MTL framework to enhance SOH evaluation and RUL prediction.

### 2.2. The Proposed Model

In the MTL model, the shared module (i.e., the encoder integrating the Transformer and 1DCNN network structure) extracts key features from the respective domain. In contrast, the designated block (i.e., the specific task layer) focuses on performing individual tasks. MTL models for RUL prediction in mechanical systems commonly employ recurrent architectures like RNN, LSTM, GRU, and BiGRU, known for their effectiveness in capturing high-dimensional and temporal dependencies in condition monitoring data. To further improve deep learning model performance, particularly in addressing local dependencies, recent advancements have incorporated attention mechanisms into these architectures [23]. Nevertheless, Transformers and their corresponding variants, such as Informer [24], have attempted to build predictive models directly using attention mechanisms, avoiding issues such as sequential computation inherent in recurrent networks. While the feature extraction method is undeniably vital, the source of feature inputs also plays a key role in determining prediction accuracy. To improve model generalization and enhance prediction accuracy through better feature representation, a low-rank transformer and 1D-CNN were introduced as the backbone framework for a multi-input MTL structure.

Additionally, in the present study, the SOH evaluation task was regarded as an auxiliary task, employing a multi-step prediction classification method to further enrich the task types within the MTL model. Under the MTL framework, SOH evaluation and RUL prediction provide equipment managers with multidimensional SOH information, enabling a comprehensive assessment of mechanical system operating conditions from various perspectives, aiding predictive maintenance, ensuring equipment reliability, and offering essential guidance for maintenance decisions.

**Figure 1:**
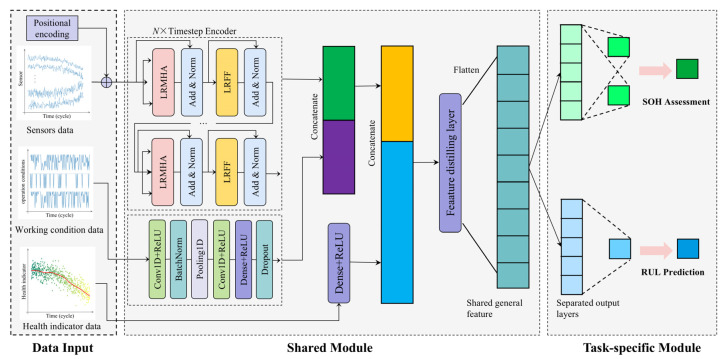
MCI-1DC+LRT model framework based on MTL.

The MTL structure was designed using a Transformer and 1D-CNN as its backbone. Figure 1 presents an overview of the proposed MTL method for implementing multi-condition SOH evaluation and RUL prediction. The proposed MTL model consists of three main modules:

Module 1: Multi-input data module. This module includes three types of data inputs: high-dimensional sensor data, operating condition data, and health indicator data.

Module 2: Multi-feature fusion shared layer module. The corresponding input data is divided into time windows, and sensor data is embedded with positional information before being fed into a shared Transformer-based encoder to generate concatenated feature maps. Condition data is input into a shared 1DCNN neural network to generate features closely related to condition variations. Degradation data from health indicators is directly connected to the features extracted from the fusion of the first two inputs and processed through a fully connected layer to extract comprehensive features.

Module 3: Task-specific module. This module is designed to implement two tasks. Task A, focused on SOH evaluation, utilizes the feature maps extracted from the shared layer as input and produces SOH evaluation results through its task-specific layer. Task B, dedicated to RUL prediction, also leverages the features from the shared layer as input and generates RUL prediction outcomes through its respective task-specific layer.

#### 2.2.1. Multi-Input Data Module

In the multi-input data module, various types of data from different sources need to be handled, including high-dimensional sensor data, operating condition data, and health indicator data. To fully utilize these different types of data, data processing needs to be performed according to the corresponding network structure. Firstly, for high-dimensional sensor data $X_{S}^{N}=\left\{x_{S}^{1},x_{S}^{2},x_{S}^{3},\ldots ,x_{S}^{t},\ldots ,x_{S}^{T}\right\}\in R^{T\times N}$, where T represents the length of the sensor time series and $X_{S}^{N}$ denotes the feature dimension of the sensor data. Sensor data serves as the input source for the Transformer network. Unlike traditional sequential input architectures such as RNN, LSTM, or GRU, the T ransformer employs a positional encoding layer to assign unique encoding values to input data at different time steps. This mechanism enables the model to effectively capture sequentiality and temporal dependency patterns within the data [25]. The definition of the positional encoding layer is as follows:

(1) $${Position}_{\left(i,t\right)}=\left\{\begin{array}{c} sin\left(\frac{t}{10000^{\frac{2i}{d}}}\right),i=2k\;\;\;\;\;\;\;\;\\ cos\left(\frac{t}{10000^{\frac{2i}{d}}}\right),i=2k+1 \end{array}\;\right.$$

where t represents the time steps of the input data; ${Position}_{\left(i,t\right)}$ is the positional encoding of time step t; d denotes the dimension of the position vector; and i represents the feature dimension of the data. Thus, for any given time series data length l, ${Position}_{\left(i,t+l\right)}$ and ${Position}_{\left(i,t\right)}$ exhibit a linear relationship, as shown in Equation (2). Therefore, the model can easily learn the relationships between positions and preserve local context.

(2) $$X_{t}^{\left(P\right)}=X_{\left(i,t\right)}^{\left(s\right)}+Position\left(X_{\left(i,t\right)}^{\left(s\right)}\right)$$

where $X_{t}^{\left(P\right)}$ is the output vector of time step t in the time series after positional encoding and $X_{\left(i,t\right)}^{\left(s\right)}$ is the input vector of sensor data at time step t.

Secondly, the operating condition data $X_{C}^{N}=\left\{x_{C}^{1},x_{C}^{2},x_{C}^{3},\ldots ,x_{C}^{t},\ldots ,x_{C}^{T}\right\}\in R^{T\times N}$ serves as the input data source for the 1DCNN network, and the health indicator data $X_{HI}=\left\{{HI}_{1},{HI}_{2},\ldots ,{HI}_{T}\right\}$, after conventional processing, can be directly fed into the corresponding network layer for feature extraction and fusion.

#### 2.2.2. Multi-Feature Fusion Shared Layer Module

The shared layer module employs a multi-input approach to achieve feature diversity. In mechanical systems operating under complex conditions, the collected data typically comprises two main types: operating condition data and system operation data under corresponding conditions. Most existing studies [26,27,28] fuse these two data types prior to input and then extract features using various models, a method that has shown strong feature extraction performance. However, this fusion method significantly increases the complexity of the feature space. To address this, an independent feature extraction strategy was adopted in the present study. For system operation data, the Transformer architecture is utilized to extract multidimensional sensor features. To reduce the parameter count and enhance computational efficiency during the Transformer extraction process, a low-rank method is integrated into the Transformer structure. For operating condition data, a 1D-CNN is employed for feature extraction. Additionally, to further enhance the representational power of extracted features, health indicator data is incorporated as a third input. Health indicator data [29], a key parameter reflecting system degradation, plays a crucial role in SOH evaluation and RUL prediction. The method for constructing health indicators has been completed in previous research by the present authors [30]. A detailed description of the implementation of each component in the shared layer module is presented as follows.

##### Sensor Feature Extraction Using Low-Rank Transformer

In the MTL task, a multi-structured neural network was integrated into the shared layer to enhance the expressive power of extracted features. However, this integration significantly increases the computational load during training, particularly within the Transformer network structure. To address this issue and strike a balance between computational efficiency and feature representation capability, a low-rank method was incorporated into the Transformer network for sensor feature extraction.

**Figure 2:**
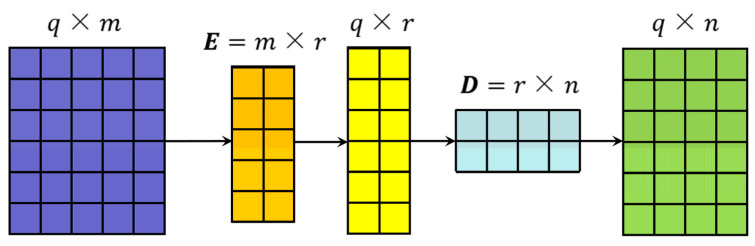
LED process.

Low-rank matrix decomposition was adopted in the linear layers for sensor input data in the Transformer model. The linear layer with low-rank matrix decomposition is referred to as the linear encoder-decoder (LED) unit [31]. As shown in Figure 2, the LED decomposition requires a parameter matrix $W\in R^{m\times n}$ in the original linear layer for sensor linear processing, which involves m×n parameters. However, with the introduction of low-rank matrices, only two smaller matrices, $E\in R^{m\times r}$ and $D\in R^{r\times n}$, are needed to approximate the parameter matrix, resulting in W≈E×D. E and D require $r\left(m+n\right)$ parameters during computation. If the rank r used in the low-rank decomposition is set to a value much smaller than parameters m and n, the parameter quantities in E and D are significantly smaller than those in W.

**Figure 3:**
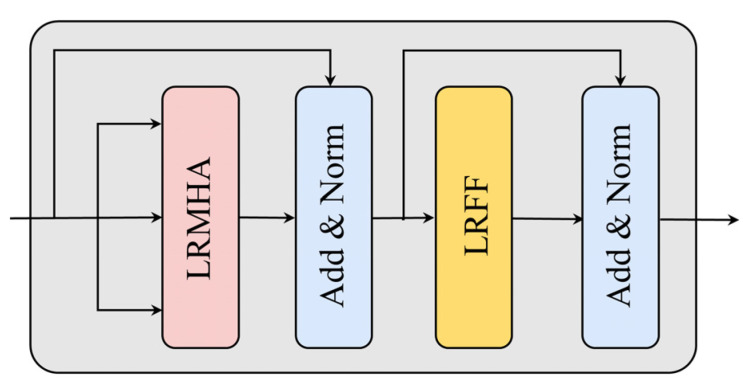
Single time step encoder.

To selectively prioritize important time step data in the sensor data, N time step encoders are used in the Transformer. The structure of a single time step encoder is shown in Figure 3, primarily composed of low-rank multi-head attention (LRMHA), low-rank feedforward neural network (LRFF), residual connections, and normalization layers.

In LRMHA, the core structure comprises stacked self-attention mechanisms applied across multiple temporal steps at varying spatial scales. This design enables the model to dynamically identify and learn the significance of each temporal step in the input data and adjust the representation weights for these steps accordingly. The self-attention mechanism evaluates the relationships among temporal steps in the input sequence, assigning differing importance levels to each step based on learned weight coefficients. The computation process for this mechanism is described by Equation (3):

(3) $$Attention\left(Q_{t},K_{t},V_{t}\right)=softmax\left(\frac{Q_{t}{\left(K_{t}\right)}^{T}}{\sqrt{d_{t}}}V_{t}\right),\left\{\begin{array}{c} Q_{t}=X_{t}^{\left(P\right)}W_{t}^{q},\;W_{t}^{q}\in R^{d_{m}\times d_{q}}\\ K_{t}=X_{t}^{\left(P\right)}W_{t}^{k},\;W_{t}^{k}\in R^{d_{m}\times d_{k}}\\ V_{t}=X_{t}^{\left(P\right)}W_{t}^{v},\;W_{t}^{v}\in R^{d_{m}\times d_{v}} \end{array}\right.$$

where $Q_{t}$, $K_{t}$, and $V_{t}$ represent the query matrix, key matrix, and value matrix of the input data, respectively; $W_{t}^{q}$, $W_{t}^{k}$, and $W_{t}^{v}$ are the corresponding weight training parameters; $d_{t}$ is a scaling factor to ensure gradient stability; $d_{m}$ represents the feature dimension of the hidden layer in the model; and $d_{q}$, $d_{k}$, and $d_{v}$ are the feature vector dimensions corresponding to the inputs $Q_{t}$, $K_{t}$*,* and $V_{t}$, respectively.

**Figure 4:**
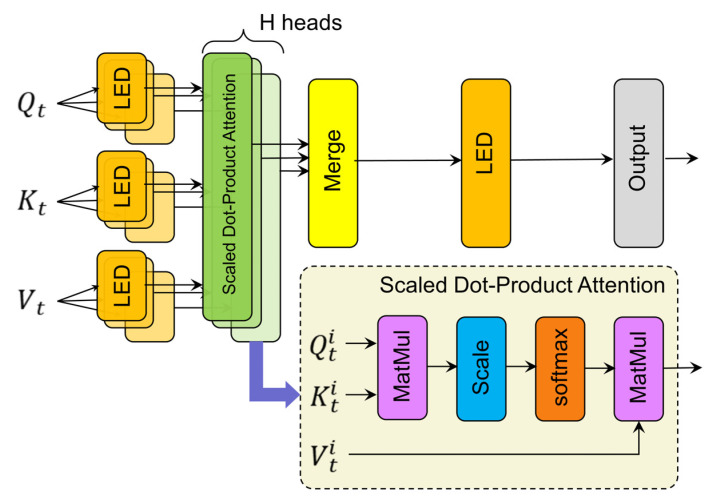
Low-rank multi-head temporal attention mechanism.

As shown in Figure 4, the LRMHA mechanism achieves subspace mapping of $Q_{t}$, $K_{t}$, and $V_{t}$ through LED. This process involves projecting $Q_{t}$, $K_{t}$, and $V_{t}$ onto discrete representative subspaces, with each subspace corresponding to a different feature extraction head. Through this mapping, the model computes the internal correlations of temporal steps in parallel across multiple self-attention heads. The outputs from these self-attention heads are then concatenated and processed through an LED layer to produce the final response of the attention mechanism. This structural design significantly enhances the representational capacity of the low-rank transformer model, enabling it to effectively capture both short- and long-range temporal dependencies in sensor data while substantially improving computational efficiency. The mathematical formulations for the output of the LRMHA mechanism are provided in Equations (4) and (5):

(4) $${hd}_{t}^{i}=Attention\left(Q_{t}E_{q}^{i}D_{q}^{i},K_{t}E_{k}^{i}D_{k}^{i},V_{t}E_{v}^{i}D_{v}^{i}\right),\left\{\begin{array}{c} Q_{t}^{i}=Q_{t}E_{q}^{i}D_{q}^{i}\\ K_{t}^{i}=K_{t}E_{k}^{i}D_{k}^{i}\\ V_{t}^{i}=V_{t}E_{v}^{i}D_{v}^{i} \end{array}\right.$$

(5) $$X_{LRMHA}=Concat\left({hd}_{t}^{1},{hd}_{t}^{2},\ldots ,{hd}_{t}^{i},\ldots ,{hd}_{t}^{H}\right)E_{o}D_{o}$$

where ${hd}_{t}^{i}$ represents the output of the self-attention mechanism at the i-th time step; H is the number of heads; $X_{LRMHA}$ represents the output features of LRMHA; Concat represents the concatenation operation across multiple heads; and $E_{q}^{i}$, $E_{k}^{i}$, $E_{v}^{i}$, $E_{o}$, and $D_{q}^{i}$, $D_{k}^{i}$, $D_{v}^{i}$, $D_{o}$ represent the address encoder matrices for query projection, key projection, value projection, and output projection, respectively.

In the time step encoder, the incorporation of residual connections allows input signals to bypass the LRMHA module and propagate directly to deeper network layers, forming shortcuts that simplify the gradient backpropagation path. This design ensures smoother gradient flow throughout the network, mitigating the vanishing gradient problem and accelerating model convergence. Additionally, the time step encoder includes layer normalization, which standardizes the output of each sublayer, stabilizing the learning process and improving data distribution. By normalizing outputs, layer normalization enhances the model’s sensitivity to individual time steps during training and reduces the impact of internal covariate shifts. These structural enhancements improve the model’s capability to represent complex temporal relationships and provide stronger generalization performance. The mathematical formulation of the output features is expressed as follows:

(6) $$X_{AN}=LayerNorm\left(X_{t}+X_{LRMHA}\right)$$

where $LayerNorm\left(\cdot \right)$ represents the layer normalization operation.

**Figure 5:**
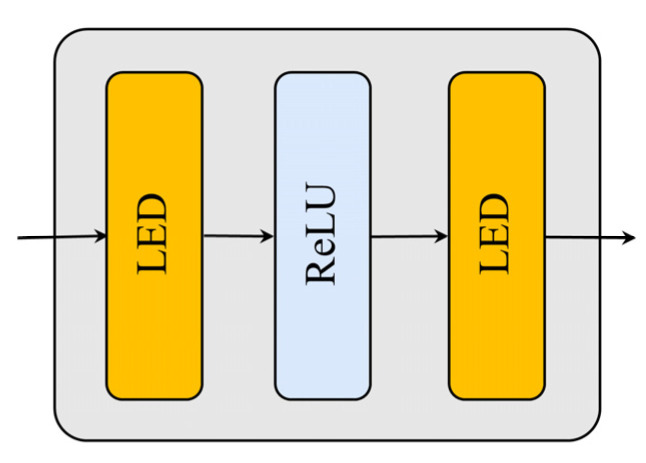
LRFF structure.

Subsequently, the LRFF network is utilized to enhance the model’s capability to represent nonlinear features while simultaneously improving the computational efficiency of feature processing at the corresponding level, as illustrated in Figure 5. The LRFF architecture is composed of two LED units, with a ReLU activation function applied between them. This design enables effective nonlinear transformation and feature representation. The output of this architecture can be mathematically expressed as:

(7) $$X_{LRFF}=max\left(0,X_{AN}E_{LR1}D_{LR1}\right)E_{LR2}D_{LR2}$$

where $X_{LRFF}$ represents the feature output of LRFF after passing through this layer; $max\left(\cdot \right)$ represents the nonlinear activation function; and $E_{LR1}$, $D_{LR1}$, and $E_{LR2}$, $D_{LR2}$ are the low-rank encoder-decoder matrices for the first and second LED units, respectively. Moreover, similar to traditional Transformer models, LRFF incorporates residual connections and normalization layer mechanisms, linking the input sequence $X_{AN}$ to the output of the second LED unit to mitigate the vanishing gradient problem. The connection output is expressed as:

(8) $$X_{AN}^{\prime }=LayerNorm\left(X_{LRFF}+X_{AN}\right)$$

where $X_{AN}^{\prime }$ represents the final output result in the time step encoder.

##### Multi-Condition Feature Extraction Using 1D-CNN

**Figure 6:**
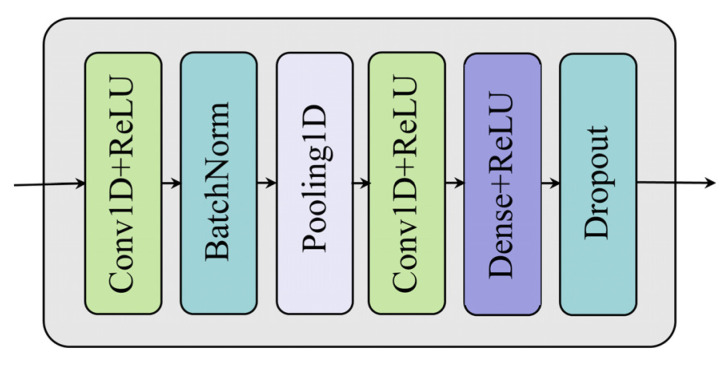
1D-CNN network architecture.

For multi-condition feature extraction, identifying corresponding features allows for more precise detection of performance variations in mechanical equipment operating under diverse conditions. CNNs, a specialized type of neural network, are well-suited for this purpose. CNNs process data through multiple convolutional stages, making them highly effective for feature extraction in both image [32,33] and sequential data [34,35] applications. To capture distinct operating condition features, a 1D-CNN network model was constructed in the shared layers of the MTL. The 1D-CNN architecture learns local patterns of one-dimensional multi-condition data through alternating convolution and pooling operations, which are subsequently fused with sensor data features. As shown in Figure 6, this network architecture primarily consists of two convolutional layers, a batch normalization layer, pooling layers, dense layers, and a dropout layer. The batch normalization layer is incorporated after the convolutional layers to stabilize the training process by normalizing feature distributions, while pooling layers are used to adjust the dimensionality of the extracted operating condition features. Dropout is applied to prevent overfitting and enhance the generalization capability of the network. The convolutional process in this architecture is mathematically represented as follows:

(9) $$X_{\left(1,i\right)}=max\left(0,X_{\left(i,t\right)}^{\left(C\right)}\times f_{\left(1,i\right)}+b_{\left(1,i\right)}\right)$$

(10) $$X_{\left(2,i\right)}=max\left(0,X_{\left(1,i\right)}\times f_{\left(2,i\right)}+b_{\left(2,i\right)}\right)$$

(11) $$X_{O}^{C}=max\left(0,W_{\left(O,i\right)}\times X_{\left(2,i\right)}+b_{\left(O,i\right)}\right)$$

where $X_{\left(i,t\right)}^{\left(C\right)}$ is the input operating condition data; $f_{\left(1,i\right)}$ and $b_{\left(1,i\right)}$ denote the convolution kernel and bias of the first convolution process; $X_{\left(1,i\right)}$ represents the output features of the first convolutional layer; $f_{\left(2,i\right)}$ and $b_{\left(2,i\right)}$ represent the convolution kernel and bias of the second convolution process; $X_{\left(2,i\right)}$ represents the output features of the second convolutional layer; $W_{\left(O,i\right)}$ and $b_{\left(O,i\right)}$ represent the weights and biases of the Dense layer; and $X_{O}^{C}$ represents the final output features of the 1D-CNN network.

##### Fusion of Health Indicators and Features

To reduce information redundancy, enhance the diversity of useful information, and simultaneously capture features containing degradation information, a fusion of the extracted features is performed. As depicted in Figure 1, the extracted sensor features and multi-condition data features represent the behavioral characteristics and response patterns of the equipment under various operating conditions. These two types of features are concatenated through a cascading fusion operation to form a new feature vector $X_{TC}$. For the health indicator feature vector, in previous research methods by the present authors, the extracted health indicator vector already included the degradation information of the equipment. A network consisting of a dense layer and a ReLU activation function is utilized to adjust the dimensions of the health indicators. The adjusted feature vector $X_{HI}$ is then concatenated with feature vector $X_{TC}$, forming a further fused feature vector $X_{TCHI}$. To enhance feature representation, a feature distillation layer was established, which consists of convolutional layers and a max pooling layer. This strategy can effectively preserve the key features and accelerate the computation process. The distilled features are flattened through a flatten layer, resulting in the final vector that prepares for the subsequent SOH assessment and RUL prediction tasks. The mathematical expression of this process is as follows:

(12) $$X_{TC}=\left[X_{AN}^{\prime \left(1\right)},\ldots ,X_{AN}^{\prime \left(d_{T}\right)},X_{O}^{C\left(1\right)},\ldots {,X}_{O}^{C\left(d_{C}\right)}\right]$$

(13) $$X_{HI}=max\left(0,W_{hi}\times X_{hi}+b_{hi}\right)$$

(14) $$X_{TCHI}=\left[X_{TC}^{\left(1\right)},\ldots ,X_{TC}^{\left(d_{TC}\right)},X_{HI}^{\left(1\right)},\ldots {,X}_{HI}^{\left(d_{HI}\right)}\right]$$

(15) $$X_{O}=MaxPool\left(Conv\left(X_{TCHI}\right)\right)$$

where $X_{TC}$ is the output feature vector obtained by concatenating the feature vectors extracted by the low-rank transformer and the 1D-CNN; $d_{T}$ and $d_{C}$ represent the corresponding dimensions of the feature vectors; $X_{HI}$ represents the output feature vector of the health indicator after passing through the dense layer and activation function; $W_{hi}$ and $b_{hi}$ are the weights and biases of the dense layer for the health indicator; $X_{hi}$ represents the sequence data of the health indicator; $X_{TCHI}$ denotes the output feature vector resulting from the concatenation of the feature vector $X_{TC}$ and feature vector $X_{HI}$; $d_{TC}$ and $d_{HI}$ correspond to the feature dimensions of the feature vectors $X_{TC}$ and $X_{HI}$, respectively; $X_{O}$ is the feature output vector from the feature fusion layer; and MaxPool denotes the max pooling layer.

#### 2.2.3. SOH and RUL Modules

In mechanical systems, the SOH evaluation method enables real-time monitoring of system health, offering an early warning mechanism for both initial and severe faults. This capability is crucial for supporting predictive maintenance strategies that are guided by the health condition of the equipment. Such strategies depend on accurately diagnosing the current state of the mechanical system. Unlike RUL prediction, which provides a long-term forecast of equipment health under uncertain future conditions, SOH evaluation delivers a more immediate and generalized assessment of the system’s operational state at specific moments. Together, SOH evaluation and RUL prediction complement each other by addressing short- and long-term aspects of equipment health.

The SOH module and the RUL module represent the specific task layers of this model. This section functions as the final output module of the model, possessing an independent network layer structure. The shared layer utilizes hidden parameters to extract general features from the mechanical system, serving as a foundation for multiple tasks. In contrast, the output layer within the specific task layer maintains its own dedicated parameters, focusing on performing specialized tasks, such as SOH evaluation or RUL prediction.

**Figure 7:**
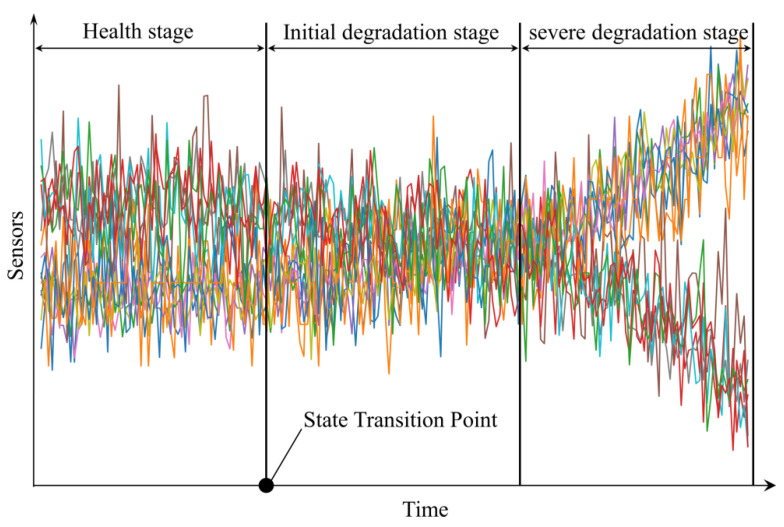
Health stages of mechanical systems.

In certain task-specific output scenarios, the RUL variable is classified as an interval-type variable, whereas the SOH serves as an ordinal target. As depicted in Figure 7, it was hypothesized that a mechanical system experiences three stages during operation, where 0 represents healthy, 1 represents degraded, and 2 represents unhealthy (k = 0, 1, 2). During the healthy phase, the mechanical system operates normally, with no observable upward or downward trends in the sensor data oscillations. During the initial degradation phase, which began at the onset of early fault detection, upward and downward trends in the sensor data became discernible. In the advanced degradation stage, these trends were more prominently pronounced. To encode these state transitions for integration into a neural network, two binary classifiers and two dummy variables were utilized. The target vectors for the health states—healthy, degraded, and unhealthy—were encoded as [0, 0], [1, 0], and [1, 1], respectively. The multi-task model was trained to predict these binary targets. During the testing phase, test samples were sequentially ranked by scanning from the first classifier to the second classifier. Each sample was associated with two probabilities. The first probability was determined using the health indicator value at the sample’s state transition point (STP) as the threshold for identifying the initial degradation phase. The STP represented abrupt changes, critical transitions, or pivotal events within the data, making its identification crucial for analyzing the progression from a healthy state to a faulty state [36]. The second threshold was determined based on the health indicator $h_{i}=0.5$ [37]. When the first probability value surpassed the health indicator value corresponding to the STP, it was set to 1; otherwise, it was set to 0. If the first probability were 1, the second probability was evaluated in the same manner. The process of predicting SOH and RUL is represented as follows:

(16) $${\hat{O}}_{SOH}\left(X_{O}\right)=\left(I_{\left\{C_{1}\left(X_{O};\theta_{1}\right)≻h_{STP}\right\}},I_{\left\{C_{2}\left(X_{O};\theta_{2}\right)≻h_{i}\right\}}\right)$$

(17) $${\hat{O}}_{RUL}\left(X_{O}\right)=Y\left(X_{O},\theta_{rul}\right)$$

where ${\hat{O}}_{SOH}\left(X_{O}\right)$ represents the output result of health status prediction; $X_{O}$ is the output feature of the shared module; $C_{1}$ and $C_{2}$ denote the classifier functions of two independent neural networks, both of which take $X_{O}$ as input features and combine parameters $\theta_{1}$ and $\theta_{2}$ to generate prediction probabilities of corresponding dimensions; $h_{STP}$ corresponds to the health indicator value based on the STP point; $I_{\left\{\cdot \right\}}$ is an indicator function that outputs 1 when the internal condition is true and 0 otherwise; ${\hat{O}}_{RUL}\left(X_{O}\right)$ represents the output result of RUL prediction; and Y and $\theta_{rul}$ are the RUL predictor and its parameters, respectively.

### 2.3. Loss Function and Network Optimization

In the MTL model, all network parameters were trained through joint optimization to simultaneously enhance task generalization and improve single-task model performance. The weight configuration of the cross-task joint loss function played a critical role in determining overall model performance. During optimization, the parameters in the shared module, acting as the globally shared component, were dynamically updated to capture general features of mechanical system degradation, while the task-specific module parameters focused on learning independent features relevant to their respective tasks. Each task’s loss function directly influenced the performance of its model and indirectly affected other tasks through its impact on the shared parameters. To address these interdependencies and optimize performance, a homoscedastic uncertainty-based method [38] was employed to dynamically adjust the weights of the joint loss function, adapting to the inherent uncertainties of different tasks and ensuring balanced optimization across the model.

In the present study, the SOH evaluation and RUL prediction tasks correspond to multi-class classification and regression problems, respectively. For the SOH evaluation task, the evaluation results and gradients are calculated using the SoftMax function, and the model’s network parameters are optimized using the cross-entropy loss function, which is expressed as follows:

(18) $$L_{SOH}\left(\theta \right)=\frac{1}{N}\sum_{i=1}^{N}\sum_{r=1}^{2}\left\{H_{i}^{r}log{\hat{H}}_{i}^{r}+\left(1-H_{i}^{r}\right)log\left(1-{\hat{H}}_{i}^{r}\right)\right\}$$

where N represents the number of samples in the time series data; $L_{SOH}$ is the loss information for SOH in the combination of two binary classifiers; and ${\hat{H}}_{i}^{r}$ is the true health state of the ith sample in category r.

The objective of the RUL prediction task is to generate the narrowest possible prediction interval (PI) while maintaining the required confidence level $\left(1-\alpha \right)$. Therefore, in the present study, an interval loss function (PIVEN) based on specific value predictions was employed to optimize the network model [39]. The loss function in the RUL prediction task simultaneously considers the losses of the PI and the predicted RUL values.

(19) $$CMPIW=\frac{1}{\sum_{i=1}^{N}k_{i}}\sum_{i=1}^{N}\left({\hat{y}}_{j,i}^{u}-{\hat{y}}_{j,i}^{l}\right)\times k_{i}\;,\;k_{i}=\left\{\begin{array}{c} 1,y_{j,i}\in \left[{\hat{y}}_{j,i}^{l},{\hat{y}}_{j,i}^{u}\right]\\ 0,y_{j,i}\notin \left[{\hat{y}}_{j,i}^{l},{\hat{y}}_{j,i}^{u}\right] \end{array}\right.$$

(20) $$L_{PI}\left(\theta \right)=CMPIW+\sqrt{N}\times \lambda \times max{\left(0,1-\alpha -p\right)}^{2}$$

(21) $$L_{PV}\left(\theta \right)=\frac{1}{N}\sum_{t=1}^{N}e_{i},e_{i}=\left\{\begin{array}{c} \rho \times {\left({\hat{y}}_{j,i}-y_{j,i}\right)}^{2},\;\;\;\;\;\;\;\;\;\;\;\;\;\;\;\;\;\left({\hat{y}}_{j,i}-y_{j,i}\right)>0\\ \left(1-\rho \right)\times {\left({\hat{y}}_{j,i}-y_{j,i}\right)}^{2},\;\;\;\;\;\left({\hat{y}}_{j,i}-y_{j,i}\right)\leq 0 \end{array}\right.$$

(22) $$L_{RUL}\left(\theta \right)=\beta \times L_{PI}\left(\theta \right)+\left(1-\beta \right)\times L_{PV}\left(\theta \right)$$

where CMPIW is the average prediction width; $k_{i}$ indicates whether the true RUL value in the RUL prediction task j for ith cycles lies within the predicted interval $\left[{\hat{y}}_{j,i}^{l},{\hat{y}}_{j,i}^{u}\right]$; $L_{PI}\left(\theta \right)$ denotes the loss of the PI under parameter θ; $max{\left(0,1-\alpha -p\right)}^{2}$ is the penalty for the portion of the predicted confidence level that does not meet the standard; p denotes the confidence metric of the prediction interval; α represents the desired confidence probability; $L_{PV}\left(\theta \right)$ measures the allocation of different loss weights to early and late predictions; and $L_{RUL}\left(\theta \right)$ denotes the loss function for RUL prediction. The parameters λ, ρ, and β in Equations (19)–(22) are hyperparameters representing the weights of each component.

When handling input data with multiple different scales, it is necessary to achieve a reasonable balance between the corresponding loss functions to ensure model performance optimization. To this end, a joint loss function was constructed for the designed MTL model based on the homoscedastic uncertainty method, as shown in Equation (23).

(23) $$L\left(\theta ,\sigma_{h},\sigma_{r}\right)\approx \frac{1}{\sigma_{h}^{2}}L_{SOH}\left(\theta \right)+\frac{1}{2\sigma_{r}^{2}}L_{RUL}\left(\theta \right)+log\sigma_{h}+log\sigma_{r}$$

where $\sigma_{h}$ and $\sigma_{r}$ represent the weight parameters associated with the SOH task and the RUL task, respectively. The loss function not only balances the importance of multiple tasks but also integrates the capability to handle uncertainty, enabling the model to adaptively adjust to varying confidence levels associated with the SOH and RUL prediction tasks. Algorithm 1 outlines the method used to determine the optimal parameters based on the joint loss function within the proposed model.
**Algorithm 1.** Optimization of the proposed model using joint loss function**Input:** Input training data $X_{S}^{N}$, $X_{C}^{N}$, $X_{HI}$; multi-task model parameter θ; uncertainty parameters $\sigma_{h}$, $\sigma_{r}$; number of training rounds N; batch number $n_{b}$; total batches $N_{b}$; learning rate λ; **Output**: Optimized model parameters and uncertainty parameters θ, $\sigma_{h}$, $\sigma_{r}$. 1: **FOR** k < −0 to N **DO** 2:      **FOR** $n_{b}$ < −0 to $N_{b}$ **DO** 3:         Feed the divided data into the proposed model; 4:         Calculate the loss $L_{SOH}\left(\theta \right)$ of the SOH evaluation task; 5:         Calculate the loss $L_{RUL}\left(\theta \right)$ of the RUL prediction task; 6:         Calculate the minimum joint loss function $L\left(\theta ,\sigma_{h},\sigma_{r}\right)$ based on the logarithm mic variance; 7:         Update parameter $\theta ,\sigma_{h},\sigma_{r}$; 8:          $n_{b}\leftarrow n_{b}$ + 1; 9:      **END FOR** 10:       k←k+1; 11: **END FOR** 12: return Optimized $\theta ,\sigma_{h},\sigma_{r}$;

## 3. Experimental and Analysis

### 3.1. Dataset Introduction

**Figure 8:**
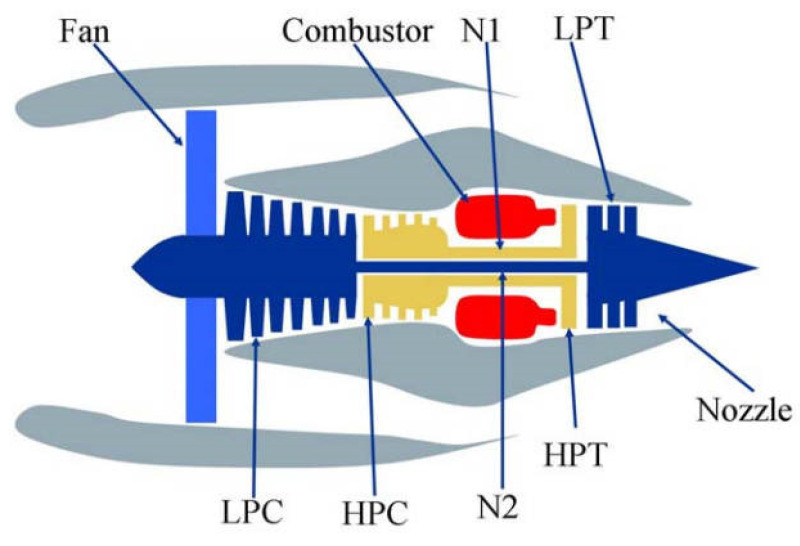
Turbine engine structure.

To validate the performance of the improved method, it was applied to a simulated degradation dataset of aircraft engines generated using the Commercial Modular Aero-Propulsion System Simulation (C-MAPSS) tool [40]. As shown in Figure 8, the data originated from a simulation model of a turbine engine structure with 90,000 pounds of thrust. The C-MAPSS model, known for its detailed fault mode designs and diverse operating conditions, is widely used in the field of RUL prediction. Its operating conditions include: (1) altitude ranging from ground level to 40,000 feet; (2) Mach number between 0 and 0.90; and (3) sea-level temperature ranging from 60 °F to 103 °F.

**Figure 9:**
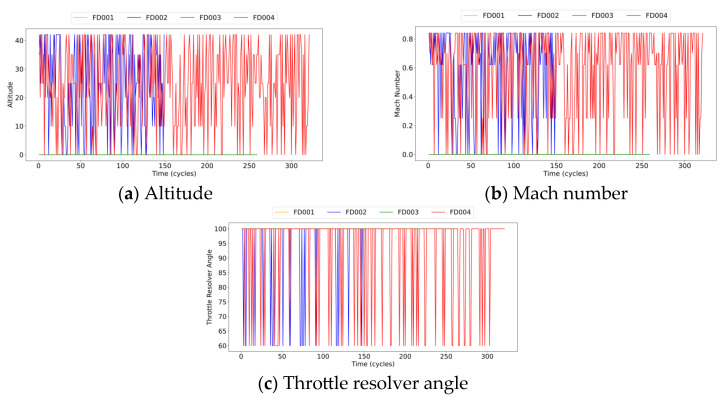
Operational data of Engine #1 in FD001–FD004.

Based on this simulation model, the C-MAPSS dataset records time series data reflecting engine performance degradation and is organized into four subsets: FD001, FD002, FD003, and FD004. Each subset comprises a training set and a test set, with the test set including RUL labels for the engines. The dataset contains 26 features, of which 21 are operational signals measured by sensors, 3 are operational environment variables (altitude, Mach number, and temperature), and the remaining 2 correspond to the engine’s unique identifier and cycle count. The specific parameters of the sensor data are detailed in Table 1. To enhance data realism, the initial wear levels of the engines vary across the dataset. The FD001 and FD003 subsets represent engine performance degradation under single operating conditions, while FD002 and FD004 capture degradation across six different operating conditions. Given the significant impact of operating condition changes on engine performance, experiments were conducted on all four subsets to evaluate the proposed model’s effectiveness in SOH assessment and RUL prediction under complex conditions and diverse fault modes. Figure 9 illustrates the variation trends of operational monitoring data for Engine #1 in the training datasets FD001 and FD002. As shown, in FD001 and FD003, the operating conditions remained stable, whereas in FD002 and FD004, parameters such as altitude, throttle angle, and Mach number fluctuated continuously. Therefore, incorporating the impact of operating condition variations into the model can significantly improve prediction accuracy and enhance the model’s applicability and reliability in real-world scenarios.

### 3.2. Data Preprocessing

In the present study, sensor data with significant degradation characteristics were selected for training and testing the multi-task model. The dataset contained monitoring data from 21 sensors, where the values of sensors 2, 3, 4, 8, 11, 13, 15, and 17 exhibited an increasing trend with operational cycles, while the values of sensors 7, 9, 12, 14, 20, and 21 decreased over operational cycles. The output of the remaining sensors remained stable, indicating that these data had no practical value for the model. Therefore, the focus of the present study was on the 14 sensors that demonstrated clear trends, along with three operational condition variables, as the core inputs for analyzing the performance of the multi-task model. Further, the monitoring values of these sensors exhibited differences in scale.

As shown in Table 2, the subsets FD002 and FD004 included six different operating conditions, but the dataset did not explicitly label the specific operating conditions of the engine in each cycle. To address this issue, a density-based spatial clustering algorithm (DBSCAN) [41] was applied to group data with identical operating conditions. Subsequently, the data were standardized based on the operating conditions. The purpose of this standardization was to eliminate the effects of dimensional differences among sensor data, ensuring consistent contributions to the model [42]. Further, the monitoring data for operating conditions were normalized, while the health indicator data had already been processed into standard-range output data in prior research. For this purpose, the common z-score standardization method [43] was employed to scale the data.

(24) $$x_{i}^{\prime }=\frac{x_{i,j}-\mu_{i,j}}{\sigma_{i,j}}$$

where i represents the sensor number; j represents the operating condition; $x_{i}^{\prime }$ indicates the normalized data for the ith sensor; $x_{i,j}$ represents the raw data of the ith sensor under the operating condition j; and $\mu_{i,j}$ and $\sigma_{i,j}$ represent the mean value and corresponding standard deviation of the ith sensor under the operating condition j, respectively. Notably, FD001 and FD003 belonged to a single operating condition. Therefore, for FD001 and FD003, the j value equaled 1 during standardization.

### 3.3. Performance Evaluation Metric

In the present study, to validate the effectiveness of the proposed multi-task model, multiple evaluation metrics were introduced for analysis. For the SOH task, accuracy (ACC) is a commonly used metric for assessing classification task precision, while the macro-averaged F1 score (macro F1) [37] is a typical comprehensive evaluation standard for multi-class tasks. Therefore, ACC and macro F1 were selected as evaluation metrics for the multi-class, multi-task model, and their specific definitions are as follows:

(25) $$ACC=\frac{TP+TN}{TP+TN+FP+FN}$$

(26) $${F1}_{mac}=\frac{2\times P_{mac}\times R_{mac}}{P_{mac}+R_{mac}}$$

where TP represents the number of correctly predicted positive classes; TN represents the number of correctly predicted negative classes; FP represents the number of incorrectly predicted positive classes; FN represents the number of incorrectly predicted negative classes; $P_{mac}$ and $R_{mac}$ represent precision and recall, respectively, which can be expressed as $P_{mac}=\frac{1}{K}\sum_{k=0}^{K-1}\frac{{TP}_{k}}{{TP}_{k}+{FP}_{K}}$ and $R_{mac}=\frac{1}{K}\sum_{k=0}^{K-1}\frac{{TP}_{k}}{{TP}_{k}+{FN}_{K}}$; and k represents the number of phases categorized as SOH. Higher values of ACC and ${F1}_{mac}$ indicate better performance of the model in the task evaluation for SOH.

For the RUL prediction task, three evaluation metrics were utilized to measure predictive performance, including RMSE, mean absolute error late phase (MAELP), and penalty score function (PSF), to assess the deviation between predicted and actual RUL values [44]. Specifically, RMSE assigns equal weight to early and late prediction errors, as shown in Equation (26). However, in practical applications, the accuracy of late-stage predictions is more critical than early-stage predictions, as errors in late predictions have a more significant impact on system performance and reliability. To address the limitations of the RMSE metric in emphasizing late-stage accuracy, the present study introduced the mean absolute error for late predictions (MAELP) and the prediction severity factor (PSF). MAELP quantifies the mean absolute error specifically for late-phase predictions, focusing on engines nearing the end of their operational lifespan. PSF, on the other hand, is an asymmetric function that imposes greater penalties on late prediction errors, thereby highlighting their critical importance. The specific definitions and formulations of these three metrics are provided below:

(27) $$RMSE=\sqrt{\frac{1}{N}\sum_{i=1}^{N}e_{i}^{2}}$$

(28) $$MAELP=\frac{1}{N^{\prime }}\sum_{i=m}^{N^{\prime }}\left|e_{i}\right|$$

(29) $${Score}_{i}=\left\{\begin{array}{c} e^{-\frac{e_{i}}{13}}-1,\;\;\;\;\;\;e_{i}<0\\ e^{\frac{e_{i}}{10}}-1,\;\;\;\;\;\;\;\;e_{i}\geq 0 \end{array}\right.$$

(30) $$PSF=\frac{1}{N}\sum_{i=1}^{N}{Score}_{i}$$

where $e_{i}={RUL}_{i}^{pred}-{RUL}_{i}^{label}$ is the difference between the predicted RUL value and the actual RUL value for the ith sample; m represents the number of cycles in the last two SOH phases for the current sample; and $N^{\prime }$ represents the starting cycle of the third SOH phase in the current sample. Among these metrics, smaller values indicate better performance of the prediction model.

### 3.4. Experimental Parameter Settings

The experiments for the proposed model were conducted on an Inter ^®^ core^TM^ i7-10700 CPU (Intel Corporation, Santa Clara, CA, USA), NVIDIA RTX3090 (NVIDIA Corporation, Santa Clara, CA, USA), Python 3.6, and Torch (1.10.0+cu113). The detailed hyperparameters of the proposed model are shown in Table 3. During training, a batch size of 256 and a sliding window size of 30 were utilized. As such, based on the number of selected sensors, the dimensions of input data were unified as follows: sensor data with dimensions 256 × 30 × 14, operational data with dimensions 256 × 30 × 3, and health indicators with dimensions 256 × 30 × 1. In the low-rank transformer, to maximize computational efficiency, the rank size was preliminarily set to half of the input dimension, that is, 7. Additionally, the Adam algorithm was adopted to optimize the parameters of the multi-task model, with the learning rate set to 1 × 10^−3^ and training epochs to 300.

### 3.5. Experimental Results

#### 3.5.1. Prediction Results of the Proposed MID-1DC+LRT Multi-Task Model

**Figure 10:**
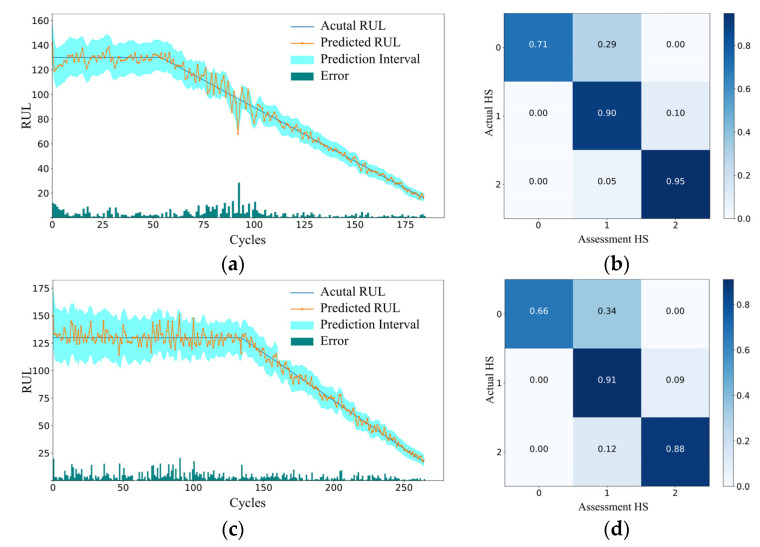

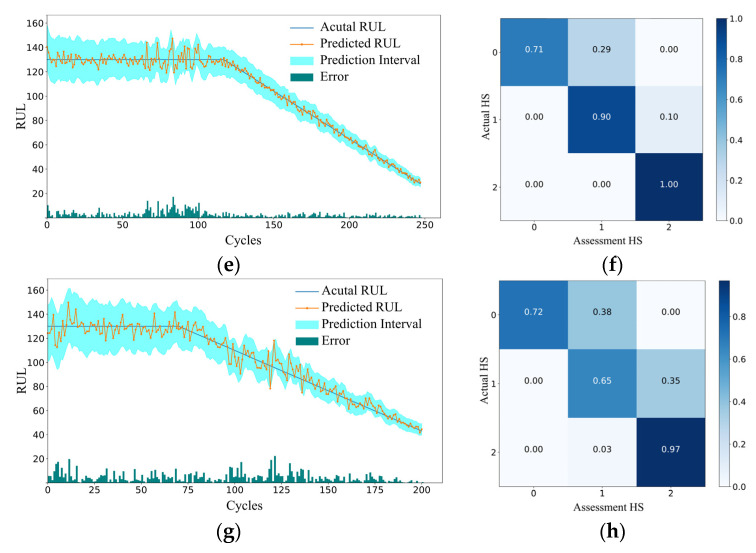
RUL predictions and SOH evaluation results of randomly selected engines in each data subset. (**a**) RUL prediction of Engine 20 in FD001; (**b**) SOH of Engine 20 in FD001; (**c**) RUL prediction of Engine 70 in FD002; (**d**) SOH of Engine 70 in FD002; (**e**) RUL prediction of Engine 100 in FD003; (**f**) SOH of Engine 100 in FD003; (**g**) RUL prediction of Engine 190 in FD004; (**h**) SOH of Engine 190 in FD004.

In the present study, a multi-input, multi-task model was proposed to predict the SOH and RUL of aircraft engines. The model employs a joint loss function approach incorporating homoscedastic uncertainty to update model parameters while also quantifying prediction uncertainty using PI. This feature enables operators to formulate effective maintenance strategies aimed at minimizing industrial production losses. During the prediction phase, forecasts were made from the first operational cycle up to the RUL values provided in the dataset’s RUL file. Figure 10 illustrates the RUL predictions and SOH evaluations of engines extracted from a subset of the C-MAPSS dataset using the proposed model. As shown in the figure, the model’s predictions closely aligned with the actual health status of the equipment in most cases, thereby validating its reliability and practical applicability.

Figure 10a,c,e,g demonstrates that the proposed method achieved strong predictive performance in RUL prediction, particularly during the fault phase, where favorable results were observed across all four datasets. Regarding prediction trends, as the number of prediction cycles increased, the predicted engine RUL gradually decreased and converged with the actual RUL. In the early stages, when the engine remained in a healthy state, the model had limited access to meaningful engine state information, leading to relatively large prediction error. Nonetheless, in some cases, prediction errors suddenly increased during the initial degradation phase. This phenomenon could be primarily attributed to the nonlinear nature of engine degradation. With limited initial state information, the model struggled to accurately capture the trend during the early accelerated degradation phase, resulting in a sudden rise in prediction error. During the fault phase, operational data exhibited pronounced anomalies, and degradation characteristics became most apparent, leading to the highest prediction accuracy and the lowest errors throughout the prediction process. Further, at a given confidence level, the PI widths become progressively narrower, with the majority of RUL predictions falling within these intervals. Notably, the PI intervals for FD001 and FD003 were significantly narrower compared to those for FD002 and FD004. This difference arose because the operating condition data in FD001 and FD003 were more uniform, simplifying the extraction of critical features and improving prediction precision.

Figure 10b,d,f,h shows the SOH evaluation results for the selected engines, illustrating the classification accuracy (ACC) probabilities derived from the confusion matrix for the evaluated and actual SOH states. These metrics were used to assess the model’s performance in predicting system SOH. From the results for the four selected engines, it is evident that the ACC for both the actual and evaluated SOH exceeded 0.65. In the healthy stage, the prediction ACC was generally lower, while in the fault stage, it improved significantly, even reaching 1.0, mirroring the trend observed in RUL prediction errors. In FD001, during the initial degradation phase, RUL prediction errors were relatively large; however, the SOH evaluation accuracy reached 0.9. This phenomenon can be attributed to the model’s uncertainty handling mechanism, which expanded the confidence interval for more challenging samples. This is particularly apparent in FD001, where the prediction interval was narrower during this stage, allowing the model to maintain high accuracy in SOH evaluation even under challenging prediction scenarios.

#### 3.5.2. Comparison with Related Work

To evaluate the effectiveness of the proposed model, its predictive performance was compared against recent related works. A comparative analysis was conducted using various deep learning algorithms from recent studies on the FD001–FD004 datasets. As summarized in Table 4, Table 5, Table 6 and Table 7, each prediction task was repeated 10 times, and the average results were taken as the final performance metrics. This approach ensured a robust and reliable comparison of the model’s capabilities against existing methodologies. The average metrics of different models across all engines in the four test datasets are represented as $\bar{RMSE}$, $\bar{MAELP}$, $\bar{PSF}$, $\bar{ACC}$, and $\bar{{F1}_{mac}}$. In the tables, the best results are highlighted in bold, while the second-best models are underlined. From Table 4, Table 5, Table 6 and Table 7, it can be observed that the proposed multi-task model outperformed other multi-task models in most cases. Specifically, the proposed model’s RUL prediction performance on the FD003 test dataset was second only to the multi-task BiGRU model. On the FD003 test dataset, the differences in various metrics between the proposed model and the multi-task BiGRU model ranged from 1.4 to 0.05. The score differences were minor, and the multi-task BiGRU model also demonstrated reasonable performance on other datasets. However, overall, the proposed model consistently outperformed the multi-task BiGRU model.

In terms of performance, it can be observed that the proposed model outperformed other comparative methods on the test datasets (except for the FD003 dataset), indicating that the proposed model can better predict the RUL of engines. The performance table illustrates the prediction performance during the later SOH stages of engines; as shown in Table 4, Table 5, Table 6 and Table 7, the proposed model performed better on most datasets. This conclusion is further supported by the analysis of performance metrics. The results indicate that the proposed multi-task model surpassed other models in the SOH evaluation task. However, similar to the RUL prediction metrics, the model performed less effectively on FD003 compared to other datasets. This is primarily because the proposed model treats operational condition data as an independent input, whereas FD003 represents a single-condition dataset, making independent operational feature extraction less impactful. Consequently, the prediction results for FD003 rely heavily on feature extraction from sensor data and health indicators. Although FD001 is also a single-condition dataset, its operational data is relatively easier to predict compared to FD003, which accounts for the proposed model’s better performance on FD001. In contrast, the results from the FD002 and FD004 datasets demonstrate that the proposed model achieved exceptional performance in both RUL and SOH predictions for multi-condition data, further validating its effectiveness in handling complex and diverse operating scenarios.

#### 3.5.3. Ablation Study

**Figure 11:**
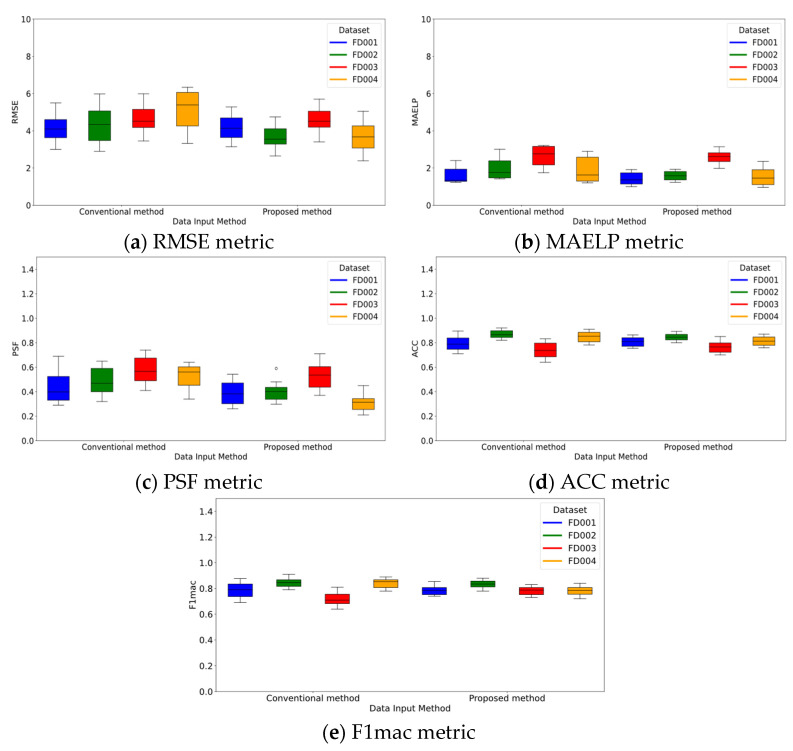
Comparative analysis of ablation experiments on input methods.

To further evaluate the proposed method’s performance on multi-condition datasets, an ablation experiment was conducted to analyze the impact of different input methods on model performance. The input data was divided into two approaches: the conventional method, which integrates condition data into sensor data, and the proposed method, which extracts condition features independently. In the conventional fusion approach, the 1D CNN network responsible for extracting condition features was removed, while other parts of the model remained unchanged. To minimize the effect of random initialization, each model configuration was run 10 times on the same dataset. Figure 11 shows a comparison of the performance of the two input methods across the four datasets. For the single-condition datasets FD001 and FD003, as shown in Figure 11, the performance of both methods was consistent across all metrics. This is because, under single-condition scenarios, variations in condition features have a limited impact, and the model’s predictions rely predominantly on operational states reflected in the sensor data. However, for the multi-condition datasets FD002 and FD004, the proposed method of independent condition feature extraction significantly outperformed the conventional fusion method in capturing condition complexity. This demonstrates the clear advantage of the proposed model in predicting under complex operating conditions. Notably, as shown in Figure 11d,e, in evaluating ACC and F1mac metrics for FD002 and FD004, the conventional fusion method slightly outperformed the condition feature separation method. This could be due to the high complexity of classification boundaries in multi-condition tasks. By integrating condition data, the conventional method increased data dimensionality during feature extraction, enabling the feature extractor to capture subtle differences between classes more effectively. This helps the model define classification boundaries more accurately in these specific metrics. Despite this slight advantage in classification metrics, the proposed method significantly outperformed the conventional method in RUL metrics and was only marginally inferior in SOH metrics. This indicates that the independent condition feature extraction method offers superior predictive performance in capturing condition features, emphasizing its importance in distinguishing influential factors and enhancing feature differentiation. Overall, these results highlight the critical role of condition data in prediction tasks and underscore the effectiveness of separating and independently extracting condition features for improved model performance.

Secondly, to compare the contribution of the low-rank transformer introduced into the proposed model to its computational efficiency, ablation experiment analysis was conducted. During the experiment, the low-rank sections in the proposed model were reverted to the original Transformer structure, and experiments were conducted to compare different rank sizes. In terms of parameter settings, only the parameters of the Transformer part were modified, while the parameters of other structures remained unchanged. Additionally, the multi-task BiGRU model from existing studies was included for comparative analysis. Similarly, to minimize accuracy inconsistencies caused by random initialization, the same model was run 10 times on the same dataset, and the average computation efficiency of the 10 runs was calculated. As shown in Table 8, the results indicate that, from a model perspective, the multi-task BiGRU model exhibited the highest computation efficiency, likely due to its unique GRU architecture, which features parallel computation capabilities for processing sequential data. The computation efficiency of the multi-task low-rank transformer was slightly lower than that of the multi-task BiGRU, with both achieving computation times within 13 s. In terms of the $\bar{RMSE}$ and $\bar{ACC}$ metrics, the multi-task low-rank transformer clearly outperformed the multi-task BiGRU, as confirmed in Section 3.5.2. From the perspective of rank size, selecting an appropriate rank is crucial for maximizing model performance. Three different rank sizes were analyzed, and the results indicate that when the rank equaled 7, the multi-task low-rank transformer exhibited balanced performance in terms of $\bar{RMSE}$, $\bar{ACC}$, and computation efficiency. Smaller ranks improved computation efficiency but led to declines in prediction scores and accuracy; larger ranks slightly enhanced prediction scores and accuracy but significantly reduced computation efficiency. Therefore, overall, the proposed model achieved good performance and computation efficiency when the rank was set to 7.

## 4. Conclusions

In the present study, a novel MID-1DC+LRT model in the MTL framework was proposed for SOH assessment and RUL prediction of sensor devices. The model consists of three components: the input layer, the shared layer, and the task-specific layer. In the input layer, the data is divided into sensor data, condition data, and health indicator data to extract relevant information from each. In the shared layer, a low-rank transformer and 1DCNN are employed to extract deep features from sensors and conditions, fuse these with health indicator features, and refine task-relevant shared features through a feature distillation layer. For RUL prediction, the PI method is used to predict RUL and quantify the uncertainty in the predictions. Additionally, the weight of the joint loss function between the two tasks was adaptively adjusted using a homoscedastic uncertainty method. The proposed model was compared with other deep learning models and evaluated through ablation experiments on the C-MAPSS dataset. The results show that the proposed model generally outperforms other models. However, despite the significant achievements in SOH assessment and RUL prediction, the model’s interpretability remains a challenge. In future work, the aim is to enhance the model’s interpretability to enable users and domain experts to better understand its decision-making process and results. Specifically, the plan is to incorporate feature importance analysis, decision process visualization, and interpretable model integration to improve interpretability, making the model more understandable and accepted, thereby enhancing its practical feasibility and credibility.

In addition, it is important to note that while this study used classical sinusoidal positional encoding, recent advancements in positional encoding, such as Rotary Positional Embedding and Attention with Linear Bias, have shown promising results in improving the extraction of long-range dependencies in sequences. These modern methods could potentially enhance the performance of our model in future iterations, especially in handling more complex sequential data. Exploring these approaches could be a valuable direction for future research to further refine the model’s capability in capturing long-range relationships within sensor data and improving overall predictive accuracy.

## Figures and Tables

**Table 1:** C-MAPSS simulation model parameter description.

Index	Symbol	Description	Units
1	T2	Total temperature at fan inlet	°R
2	T24	Total temperature at LPC outlet	°R
3	T30	Total temperature at HPC outlet	°R
4	T50	Total temperature at LPT outlet	°R
5	P2	Pressure at fan inlet	psia
6	P15	Total pressure in bypass-duct	psia
7	P30	Total pressure at HPC outlet	psia
8	Nf	Physical fan speed	rpm
9	Nc	Physical core speed	rpm
10	epr	Engine pressure ratio	-
11	Ps30	Static pressure at HPC outlet	rpm
12	phi	Ratio of fuel flow to Ps30	pps/psi
13	NRf	Corrected fan speed	rpm
14	NRc	Corrected core speed	rpm
15	BPR	Bypass ratio	-
16	farB	Burner fuel-air ratio	-
17	htBleed	Bleed enthalpy	-
18	Nf_dmd	Demanded fan speed	rpm
19	PCNfR_dmd	Demanded corrected fan speed	rpm
20	W31	HPT coolant bleed	lbm/s
21	W32	LPT coolant bleed	lbm/s

**Table 2:** Overview of C-MAPSS dataset characteristics.

Dataset	FD001	FD002	FD003	FD004
Working conditions	1	6	1	6
Fault modes	1	1	2	2
Number of engine samples	100	260	100	249

**Table 3:** Hyperparameter settings in the proposed models.

Module	Layers	Parameters	Value
Shared module (Sensor data)	Positional encoder	Dimension	64
	Low-rank transformer	Time step encoder	4
		Rank	7
	LRMHA	Heads number	4
		Head dimension	16
	LRFF	Feed forward dimension	256
		Dropout rate	0.1
Shared module (Working condition data)	1DCNN	Conv1D filters	64
		Kernel size	3
		Pooling size	2
	Dense+ReLU	Output dimension	128
		Dropout rate	0.1
Shared module (Health indicator data)	Dense+ReLU	Output dimension	64
Feature extraction and stitching	Feature distilling layer	Output dimension	256
Training process parameters		Batch size	256
		Learning rate λ	1 × 10^−3^
		Epochs	300

**Table 4:** Comparison of different models on FD001 test dataset.

Model	RUL Prediction Metrics			SOH Metrics	
	$\bar{RMSE}$	$\bar{MAELP}$	$\bar{PSF}$	$\bar{ACC}$	$\bar{{F1}_{mac}}$
Multi-task RNN	5.4565	2.4732	0.4905	0.7803	0.7687
Dual-task LSTM	16.3320	3.8701	6.1219	0.6461	0.6544
Multi-task BiGRU	4.3323	1.9218	0.3598	0.7121	0.7387
Multi-task -ACNN	3.9911	1.7767	0.3512	0.7939	0.7947
Multi-task-ACGAN	11.4772	2.3988	4.1541	0.6855	0.6731
The proposed model	3.9881	1.3142	0.3243	0.8216	0.8213

**Table 5:** Comparison of different models on FD002 test dataset.

Model	RUL Prediction Metrics			SOH Metrics	
	$\bar{RMSE}$	$\bar{MAELP}$	$\bar{PSF}$	$\bar{ACC}$	$\bar{{F1}_{mac}}$
Multi-task RNN	5.8968	3.6532	0.5783	0.7458	0.7633
Dual-task LSTM	23.766	5.8770	8.1264	0.6365	0.6204
Multi-task BiGRU	5.2155	2.5635	0.7588	0.6822	0.6835
Multi-task-ACNN	4.1552	1.7840	0.4582	0.7555	0.7385
Multi-task-ACGAN	13.2897	2.6645	3.4750	0.6944	0.7011
The proposed model	3.4256	1.5348	0.3582	0.8935	0.8962

**Table 6:** Comparison of different models on FD003 test dataset.

Model	RUL Prediction Metrics			SOH Metrics	
	$\bar{RMSE}$	$\bar{MAELP}$	$\bar{PSF}$	$\bar{ACC}$	$\bar{{F1}_{mac}}$
Multi-task RNN	5.2546	3.5865	0.5548	0.7254	0.7155
Dual-task LSTM	12.4353	4.1273	7.8542	0.6348	0.6324
Multi-task BiGRU	3.0187	1.3548	0.3658	0.8457	0.8402
Multi-task-ACNN	4.5612	3.4353	0.4765	0.7468	0.7437
Multi-task-ACGAN	11.5615	2.5632	2.8465	0.6925	0.6825
The proposed model	4.4574	2.5450	0.5119	0.7822	0.7861

**Table 7:** Comparison of different models on FD004 test dataset.

Model	RUL Prediction Metrics			SOH Metrics	
	$\bar{RMSE}$	$\bar{MAELP}$	$\bar{PSF}$	$\bar{ACC}$	$\bar{{F1}_{mac}}$
Multi-task RNN	5.2146	4.5816	0.5147	0.6525	0.6735
Dual-task LSTM	23.6543	11.7623	23.7236	0.6143	0.6146
Multi-task BiGRU	6.3246	3.6467	0.6487	0.7223	0.7236
Multi-task-ACNN	5.2998	3.4563	0.7544	0.6888	0.6954
Multi-task-ACGAN	18.9254	4.0875	12.4599	0.6753	0.6409
The proposed model	3.5890	1.3582	0.3075	0.8524	0.8548

**Table 8:** Comparative analysis of different models and rank sizes in terms of their scores and computational efficiency.

Model	Rank Size	$\bar{RMSE}$		$\bar{ACC}$		$\mathrm{Computational}\;\mathrm{Time}\;(\tilde{t})$	
		FD001	FD003	FD001	FD003	FD001	FD003
Multi-task BiGRU	-	4.3323	3.0187	0.7121	0.8457	8.0863	10.7546
Multi-task Transformer	-	3.9466	4.1143	0.8289	0.7603	15.8045	23.4802
Multi-task low-rank transformer	6	5.2881	6.1220	0.7165	0.6791	8.4520	10.1211
	7	3.9881	4.4574	0.8216	0.7822	9.5288	12.0649
	8	3.8799	4.4127	0.8690	0.7949	15.8931	20.4621

## Data Availability

Data available on request due to restrictions.

## Abbreviations

PHM	predictive health management
SOH	state of health
RUL	remaining useful life
MID	multi-input data
1D-CNN	1d convolutional neural network
KL	Kullback–Leibler
LRT	low-rank transformer
HI	health index
C-MAPSS	Commercial Modular Aero-Propulsion System Simulation
RMSE	root mean square error
MTL	Multi-task learning
LED	linear encoder-decoder
LRMHA	low-rank multi-head attention
LRFF	low-rank feedforward
STP	state transition point
PI	prediction interval
PIVEN	PIs with specific value prediction
CMPIW	captured mean predicted width
DBSCAN	density-based spatial clustering algorithm
ACC	Accuracy
MAELP	mean absolute error late phase
PSF	penalty score function
